# Soiltesting formula fertilization with organic fertilizer addition for target yield cannot stand long due to stem lodging of rice

**DOI:** 10.3389/fpls.2022.1091156

**Published:** 2022-12-08

**Authors:** Fucheng Zhao, Fan Li, Juan Zhou, Xiaolin Sun, Yun Wang, Liquan Jing, Junfeng Hou, Fei Bao, Guiyue Wang, Bin Chen

**Affiliations:** ^1^ Institute of Maize and Featured Upland Crops, Zhejiang Academy of Agricultural Sciences, Dongyang, China; ^2^ Jiangsu Key Laboratory of Crop Genetics and Physiology Agricultural College, Yangzhou University, Yangzhou, China; ^3^ Jiangsu Key Laboratory of Crop Cultivation and Physiology, Agricultural College, Yangzhou University, Yangzhou, China; ^4^ Jiangsu Co-Innovation Center for Modern Production Technology of Grain Crops, Agricultural College, Yangzhou University, Yangzhou, China; ^5^ Eco-Environmental Protection Research Institute, Shanghai Agricultural Academy of Sciences, Shanghai, China; ^6^ Planting Technology Extension Center of Dongyang, Dongyang, China

**Keywords:** long-term located experiment, organic fertilization, stem lodging, carbohydrates, mineral element, rice yield

## Abstract

**Introduction:**

Soil testing formula fertilization using organic fertilizer (STFFOF)could increase grain yields and protect the ecological environment but the potential risks of STFFOF remains unclear.

**Methods:**

In order to assess the risk on rice stem lodging, a STFFOF field experiment is conducted continuously for 11 years.

**Results:**

After 11 years of continuous STFFOF treatment, the stem lodging rate of rice substantially increases by 81.1%*, which completely overweigh its increase in yield. Further research found that STFFOF greatly decreases the concentration of Ca, SiO2, K, Mg, and non-structural carbohydrates in basal internodes, dramatically increases that of N, P, and weight per ear, but slightly affects the structural carbohydrates. The strong correlations imply the increasement in weight per ear, N, and P concentrations, and the significant decrease in starch in the basal internodes might directly increase the brittleness of stem internodes and further cause severe stem lodging and yield loss of rice.

**Discussion:**

Results suggest that the potential risks of rice production including stem lodging must be considered when adopting the excessive exploration mode of productivity technology of paddy fields.

## Introduction

Chemical fertilizer has greatly tapped the potential of arable land for grain production in the previous decades (Liu et al., 2020; Luo et al., 2020). However, it has also caused serious problems, including low utilization rate, remarkable fertilizer losses (Liu et al., 2020), environmental pollution, overdraft of arable land, and hardening of soil (Niu et al., 2022), thereby seriously limiting the ability of chemical fertilizers to continuously increase grain production. China had widely promoted soil-testing formula fertilization using organic fertilizer (STFFOF), which was based on the demand law of plant fertilizer, soil fertilizer supply capacity, and fertilizer utilization rate (Liu et al., 2020; Luo et al., 2020; Wang et al., 2021) for good yield and was efficient in 2013 to maintain the ecological resilience of arable land (Wang et al., 2021) and ensure the sustainable material output of fields. A number of short-term experiments have shown that STFFOF not only reduced the total phosphorus, NH_4_–N, NO_3_–N, and the amount of soil fertilizer (Wu et al., 2019; Xie et al., 2019) and effectively enhanced rice yield and agricultural economic benefit (Wang et al., 2019; Liu et al., 2020) but also improved the soil ecological environment of paddy field (Wang et al., 2021). Nonetheless, considering the huge cost and the basic national conditions of developing countries, organic fertilizer application required by STFFOF has not been widely promoted in China. Despite this, it is still considered an inevitable trend of conservation tillage in the future given its advantages (Liu et al., 2020).

Rice is a staple food and provides 20% of the daily calorie needs for more than half of the population worldwide; it is even more significant to China (Zhao et al., 2015). However, profile and risk assessments should be required when carrying out some new agricultural production activities, such as STFFOF (Wu et al., 2019). Wang et al. (2021) considered that organic fertilizers are unsuitable for long-term use due to the result of a 16-year study conducted in China; it showed that the long-term use of organic fertilizers may cause pollution of heavy metals (Wang et al., 2021). Lodging also greatly harmed the rice yield and quality, thereby completely counteracting the positive effects of most measures on rice, including molecular and culturation. Lodging is difficult to control because it is a complex process determined by many factors including varieties, weather, soil type, crop man agreement, and disease episodes (Kong et al., 2014). N enrichment in stems significantly reduced the pushing resistance of plants. N promoted vegetative growth and reduced the stem wall thickness and stem diameter, thereby resulting in the reduction of the strength of stems and the increase in stem lodging risk (Yang et al., 2009; Wu et al., 2012; Kong et al., 2014; Zhang et al., 2014b; Zhao et al., 2019; Xu et al., 2020). Differently from other arable lands, paddy fields are mostly covered by a water layer during the entire growing season of crops; therefore, the ecological effect of increasing organic fertilizer on paddy fields is better theoretically. However, this condition cannot rule out the possible risk of STFFOF on rice yield or quality, especially based on the results of long-term location tests. Hence, for a sustainable high yield of rice, this study carried out an STFFOF experiment for 11 years in paddy fields to systematically evaluate potential risks specially on rice stem lodging regarding this mode.

## Materials and methods

### Study site

The experiment was performed in the farm of the Institute of Maize and Featured Upland Crops, which is located in Dongyang (120°18′ E, 29°11′ N), Zhejiang Province, China, from 2011 to 2021. The experimental plot was of red soil with good irrigation and drainage facilities. The soil contained in the upper 20 cm 22.5 g kg^-1^ organic matter, 1.43 g kg^-1^ total N, 1.31 g kg^-1^ total P, 15.8 g kg^-1^ total K, 96.7 mg kg^-1^ soil-available N, 9.87 mg kg^-1^ soil-available P, and 83.92 mg kg^-1^ soil-available K. The pH value of the soil was 5.64.

### Experimental setup and rice cultivation

Rice seeds of Zhexiangyinzhen, a popular *indica* cultivar in this region, were sown in a nursing paddy on 20 June. At 26 days later (on 16 July), uniform rice seedlings were selected and manually transplanted into subplots that were distributed randomly in each plot. The area of each plot is 3 m × 7 m. Planting density was one seedling per hill, and the spacing of the hills was 17.9 × 30 cm, equivalent to 18.6 hills m^−2^. In brief, 5.4 g P m^-2^ [15% P_2_O_5_, Ca(H_2_PO_4_)_2_·H_2_O] and 11.2 g N m^-2^ (46% N, urea) with the control were applied as basal dressing at 1 day before transplanting. Afterward, 3.3 g N m^-2^ was applied on 28 July as the tillering fertilizer, followed by the addition of 4.9 g N m^-2^ and 12.9 g K m^-2^ on 8 August as the jointing fertilizers. Relative to that in the control, 2.1 g P m^-2^ and 285.7 g m^-2^ bio-organic fertilizer (organic content ≥45%, Kingenta Co., Ltd., China) more as the basal dressing was added into the STFFOF treatment. Pesticides and fungicides were applied when required throughout the experiment.

### Theoretical and actual yield

Five plants with no lodging per plot were measured to obtain the theoretical yield of rice. All plants in each plot, including lodging plants, were measured in terms of plant maturity to obtain the actual yield.

### Parameters regarding lodging incidence in paddy field

Parameters regarding lodging incidence in rice fields were obtained according to the method reported by Zhao et al. (2019) with a few modifications: stem lodging rate was determined as a percentage ratio of broken stems to the whole plants, excluding border plants, in mature period. The whole area of each plot equals to 391 hills were surveyed to obtain the stem lodging rate. Meanwhile, 24 representative plants in the field were selected to measure the values of plant height, ear length, and weight per ear, and the SPAD value of leaf was obtained in this stage with a chlorophyll content meter (SPAS-502PLUS, Konnica Minolta Co., Ltd., Japan). The measurements of pushing resistance per plant and pushing resistance per stem were measured using a prostrate tester (ZTS-500N, IMADA Co., Ltd., Aichi-ken, Japan) and referred to its instruction manual with some modification. Briefly, the tester was set in perpendicular direction to the plants at a 20-cm height above the soil surface, and the pushing resistance (N) was recorded when the plants inclined at 45° angle to the vertical. Six representative plants in each plot were measured, and the tiller numbers were counted. The pushing resistance per stem was calculated by dividing the pushing resistance per plant by the tiller number.

### Physical structure of stem

Five other representative plants from each plot were taken to the experimental laboratory. After having been cleaned with ultrapure water, the individual stems were separated from the plants. Ten stems were blotted dry with filter paper, and the weight of a single stem was measured. The first base internodes of up to 3 cm with sheath were separated. According to our field observation, stem breakage usually occurs at this internode. The breaking resistance of this base internode was measured using the method of Zhao et al. (2019) with a prostrate tester (YYD-1A, Hangzhou, China) in the laboratory (Zhao et al., 2019). Stem wall thickness and the smallest diameters of the basal internodes were measured by a caliper. The stem density, cross-sectional area, bending moment, and lodging index of each basal internode were calculated as follows:

Stem density = dry weight/length of the basal internode

Cross-section area of basal internode = π × (diameter of basal internode/2)^2^

Bending moment of basal internode = length × fresh weight of the plant from the lower node of basal internode up to the panicle top × 0.001 × 9.8

Lodging index = bending moment of the basal internode/breaking resistance of that internode × 100

### Biochemical traits

After the measurement of fresh samples, the basal internodes were collected, dried at 75°C, and ground into powder using a Vibration Disc Mill (TS1000, Siebtechnik GmbH, Muehlheim an der Ruhr, Germany). The powder was used to measure mineral elements (including SiO_2_, N, P, K, Ca, and Mg), cell wall, lignin, and soluble sugar and starch following the method of Zhao et al. (2019). Cellulose was also measured following the method of Suzuki et al. (2009).

### Statistical analysis

A single-factor randomized block design was employed in this experiment. Each treatment had three replications. The figures and tables were created using Excel 2021. Data analysis was performed using SPSS 26 to detect the main effects of STFFOF after lasting for 11 years in paddy fields. Treatments were compared by Duncan’s test, and differences were declared as statistically significant if *P <*0.05. Statistically significant effects were indicated as follows: **P ≤0.01, *P ≤0.05, +*P* ≤0.1.

## Results

### Yield and lodging of rice

**Figure 1:**
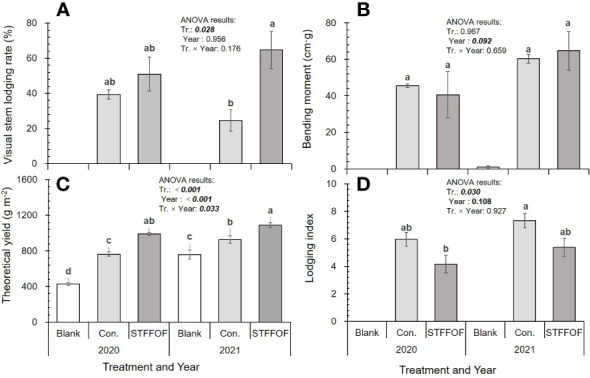
Effect of soil-testing formula fertilization using organic fertilizer (STFFOF) for 11 years on visual stem lodging rate **(A)**, bending moment **(B)**, theoretical yield **(C)**, and lodging index **(D)**. Error bars show standard errors (n = 3). Bars not sharing the same superscript letters are significantly different at P <0.05 by Duncan’s *post-hoc* comparison. ANOVA results of bending moment, lodging index, and visual stem lodging rate regarding blank, control, and STFFOF treatments are included in the figure.

**Figure 2:**
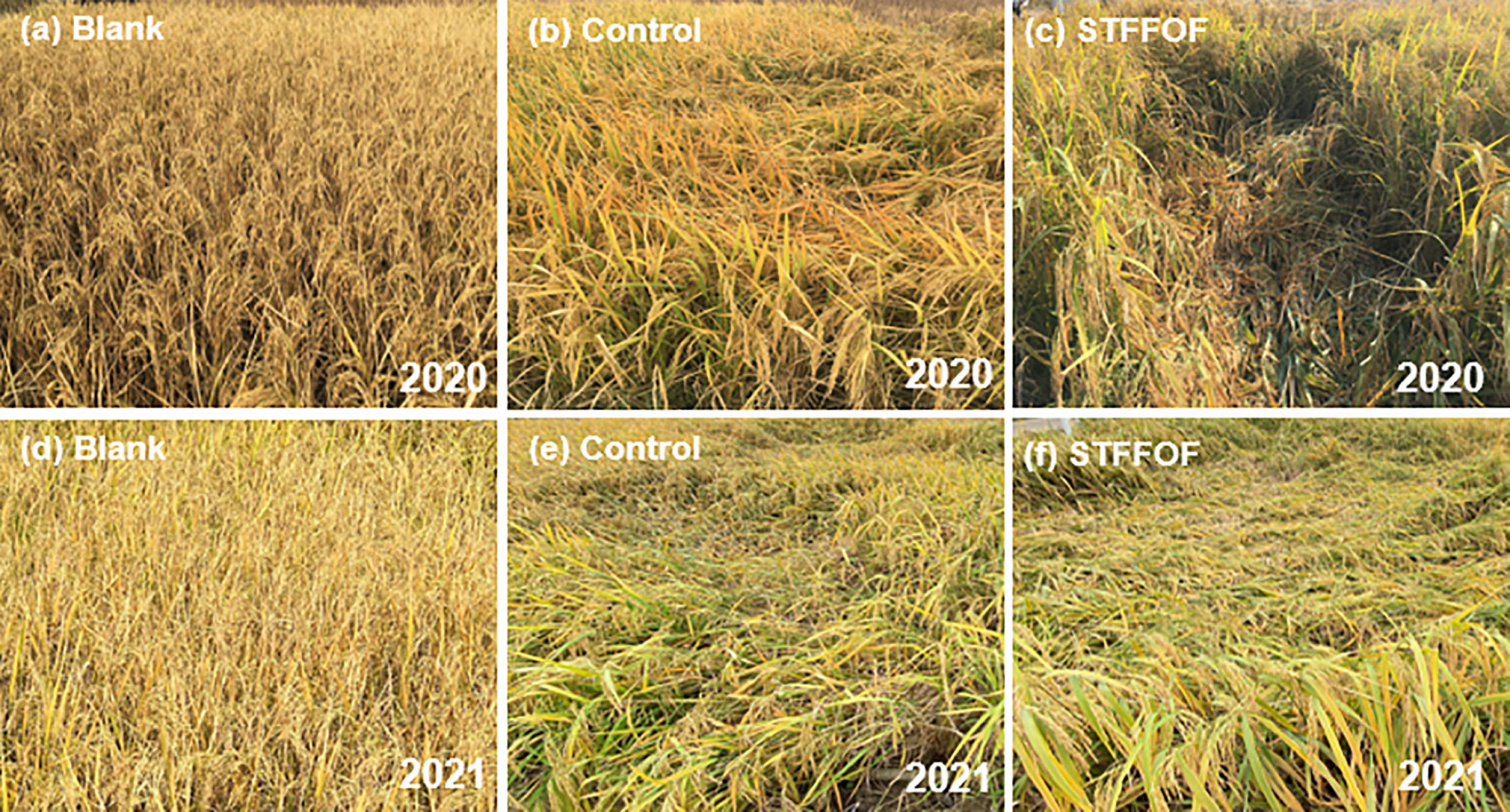
Effect of soil-testing formula fertilization using organic fertilizer (STFFOF) for 11 years on lodging incidence of rice. The representative pictures are shown for blank [no fertilizer application **(A, D)**], control **(B, E)**, and STFFOF [treatment **(C, F)**] plots during grain filling stage of rice. **(A–C)** Photographs taken on October 25, 2020. **(D–F)** Photographs taken on October 23, 2021.

**Figure 3:**
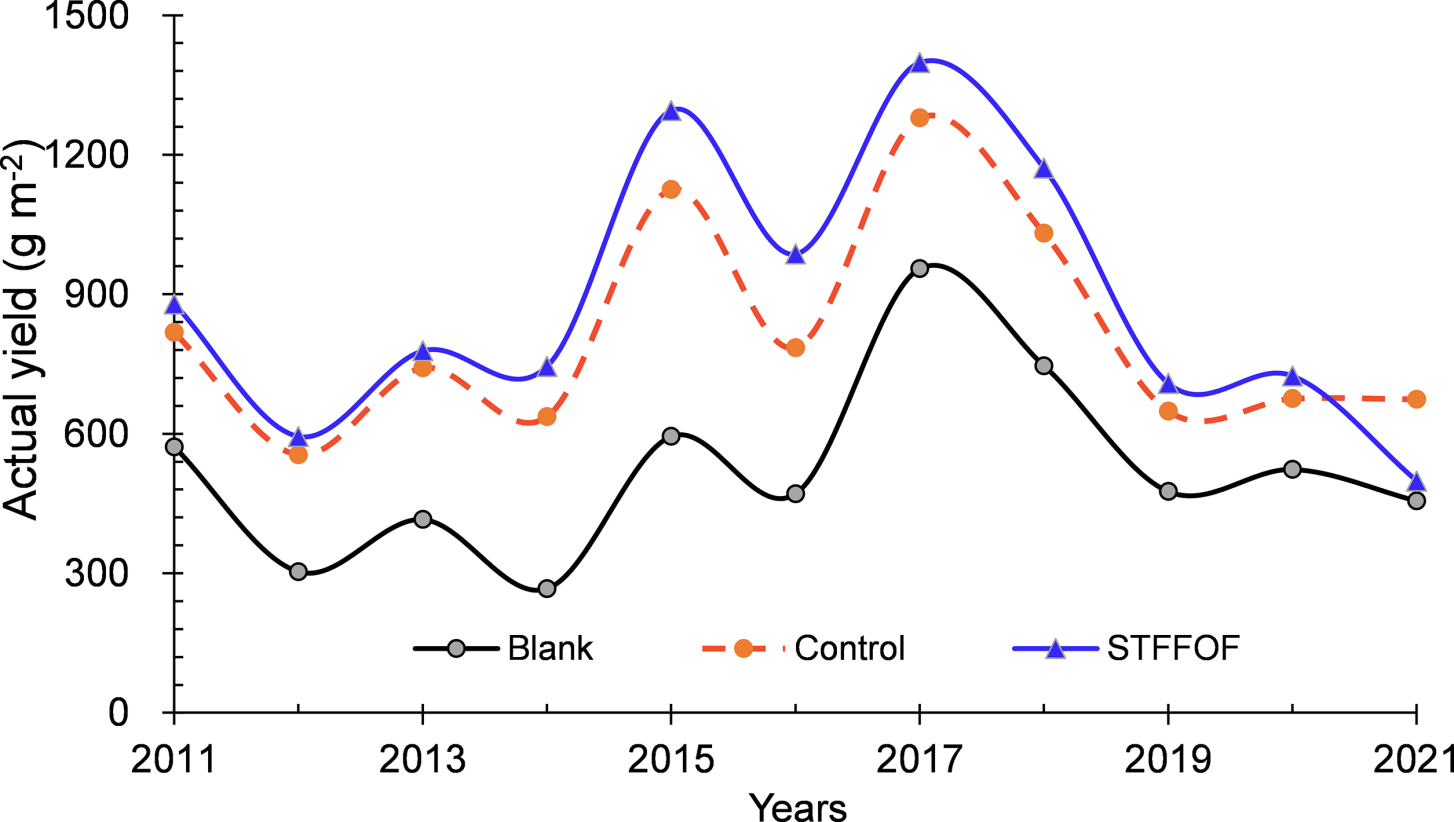
Effect of soil-testing formula fertilization using organic fertilizer for 11 years on the actual grain yield of rice.

Compared with that in the control, STFFOF treatment after 11 years reduced the lodging index of rice plants by 28.2%* averaged over 2020 and 2021 but substantially increased the stem lodging rate by 25.9 percentage points (**Figures 1A**, **2**), without a significant effect on the bending moment (**Figure 1B**). Although the theoretical yield increased by 22.9%** (**Figure 1C**), the actual yield decreased by an average of 9.5% upon STFFOF (**Figure 3**). Otherwise, no lodging in the blank treatment without fertilization application for 11 years was observed (**Figures 2A, D**). Compared with STFFOF, the yield of blank decreased slightly: the actual yield averaged for the last 2 years decreased by only 19.9% (**Figure 3**). No significant interaction effect of lodging-related indexes between treatments indicated that the effect of STFFOF on stem lodging was stable. Correlation analysis (**Table 1**) revealed the lack of a significant relationship between the stem lodging rate and lodging index or bending moment, suggesting that the visual actual stem lodging rate of paddy fields could not be estimated by the traditional lodging index under STFFOF conditions.

**Table 1:** Relationships among the physical indicators of rice stem under soil-testing formula fertilization using organic fertilizer for 11 years.

Index	SLR	LI	BM	TNP	PH	EL	WE	PRP	PRC	SPAD	WSS	BR	SD	SWT	CA
LI	-0.076														
BM	0.498+	0.624^a^													
TNP	0.546+	-0.497	0.009												
PH	-0.123	-0.013	0.150	0.143											
EL	0.189	0.218	0.090	-0.196	-0.729^b^										
WE	0.304	0.342	0.647^a^	0.343	0.408	-0.314									
PRP	-0.860^a^	0.579	-0.388	-0.775+	0.390	-0.340	-0.864^a^								
PRC	-0.818^a^	0.638	-0.253	-0.865^a^	0.446	-0.389	-0.838^a^	0.987^b^							
SPAD	0.406	-0.817^a^	-0.446	0.938^b^	-0.767+	0.284	0.358	-0.640+	-0.737						
WSS	0.544+	-0.349	0.145	0.882^b^	0.071	-0.207	0.581^a^	-0.831^a^	-0.894^a^	0.931^b^					
BR	0.695^a^	-0.539+	0.193	0.773^b^	0.356	-0.324	0.293	-0.940^b^	-0.903^a^	0.490	0.714^b^				
SD	0.654^a^	-0.574+	0.034	0.623^a^	-0.022	-0.025	0.147	-0.991^b^	-0.961^b^	0.563	0.697^a^	0.845^b^			
SWT	-0.016	-0.429	-0.223	0.123	0.292	-0.070	-0.209	-0.277	-0.288	-0.009	-0.044	0.264	0.248		
CA	0.653^a^	-0.576+	0.038	0.620^a^	-0.013	-0.029	0.147	-0.990^b^	-0.959^b^	0.555	0.693^a^	0.849^b^	10.000^b^	0.254	
CD	0.439	-0.346	0.005	0.426	-0.169	-0.252	0.133	-0.897^a^	-0.883^a^	0.641	0.387	0.403	0.444	0.143	0.438

The P values in bold show significant correlations at P <0.01. N = 6 for PRPP, PRPC, and SPAD; N = 12 for others.

SLR, stem lodging rate; LI, lodging index; BM, bending moment (cm·g); TNP, tiller number per plant; PH, plant height (cm); EL, ear length (cm); WE, weight of ear (g); PRP, pushing resistance per plant (N); PRC, pushing resistance per stem (N); WSS, weight of a single stem, apart from ear (g); BR, breaking resistance (N); SD, stem diameter (mm); SWT, stem wall thickness (mm); CA, cross-sectional area; CD, stem density (mg/cm).

a Statistically significant correlation at P < 0.05.

b Statistically significant correlation at P < 0.01.

### Parameters regarding rice plant in the field

Compared with that in the control, STFFOF averagely increased the tiller number per plant, plant height, and weight per ear by 13.7%**, 0.5%, and 14.7%*, respectively, the ear length of rice decreased by 0.6%**, and only the treatment × year interaction of the plant height presented a significant difference (**Table 2**). The SPAD increased by 21% in 2021, indicating that STFFOF enhanced the vitality of rice plants. Compared with STFFOF treatment, no significant difference in these indexes except for SPAD and tiller number per plant in the blank experiment was observed. The correlation analysis (**Table 1**) showed that the stem lodging rate was inversely related to the pushing resistance per plant and pushing resistance per stem, without evident correlation with other parameters.

**Table 2:** Effects of soil-testing formula fertilization using organic fertilizer for 11 years on the parameters regarding rice plant in the field.

Year	Treatment	Tiller number per plant	Plant height/cm	Ear length/cm	Weight per ear/g	Pushing resistance per plant/N	Pushing resistance per stem/N	SPAD
2020	Blank	9.4 ± 0.6d	100.7 ± 2.0c	27.9 ± 0.5bc	3.0 ± 0.1d			
	Con.	13.8 ± 0.3b	107.3 ± 1.4b	30.0 ± 0.1a	3.8 ± 0.1cd			
	STFFOF	15.4 ± 0.5a	110.5 ± 1.3ab	29.3 ± 0.5ab	4.1 ± 0.1c			
2021	Blank	12.0 ± 0.3c	113.9 ± 0.9a	27.6 ± 0.5c	6.3 ± 0.4ab	9.0 ± 0.3a	0.7 ± 0.0a	19.0 ± 0.2c
	Con.	13.8 ± 0.1b	112.6 ± 1.0ab	28.9 ± 0.2abc	5.9 ± 0.2b	10.1 ± 0.2a	0.7 ± 0.0a	26.5 ± 0.4b
	STFFOF	16.0 ± 0.4a	110.4 ± 1.2ab	29.2 ± 0.2ab	7.0 ± 0.3a	6.5 ± 0.4b	0.4 ± 0.0b	32.0 ± 1.6a
ANOVA results								
	Treatment	<0.001	0.165	0.007	0.013	0.001	<0.001	0.001
	Year	0.020	0.001	0.270	<0.001			
	Treatment × year	0.046	0.006	0.567	0.120			

Mean values ± standard errors (n = 3) in the same column followed by different letters are significantly different (P ≤ 0.05). The values of ANOVA results in bold show significant treatment effects at P ≤ 0.1.

Blank, no fertilizer treatment; Con., control; STFFOF, soil-testing formula fertilization using organic fertilizer.

### Physical structure of rice stem

Compared with those in the control, STFFOF increased the weight per stem and breaking resistance by an average of 65.8%** and 49.6%**, respectively, over 2020 and 2021, with only the weight per stem showing a significant difference between years (**Table 3**). The stem diameter, stem wall thickness, cross-sectional area, and stem density averagely increased by 11.4%**, 4.3%, 24.0%**, and 6.5%, respectively, upon STFFOF, of which only the treatment × year effects of weight per stem and stem density were significant. The correlation analysis (**Table 1**) showed that the stem lodging rate was only positively correlated with breaking resistance, stem diameter, and cross-sectional area under STFFOF condition for 11 years.

**Table 3:** Effects of soil-testing formula fertilization using organic fertilizer for 11 years on the physical structure of rice stem.

Year	Treatment	The weight of a single stem—apart from ear (g)	Breaking resistance (N)	Stem diameter (mm)	Stem wall thickness (mm)	Cross-sectional area	Stem density(mg/cm)
2020	Blank	26.5 ± 0.5e	12.1 ± 1.1a	5.2 ± 0.1bc	1.0 ± 0.0a	21.1 ± 0.8bc	37.5 ± 1.7bc
	Con.	39.5 ± 1.1d	7.8 ± 0.6b	5.1 ± 0.1c	1.0 ± 0.0a	20.6 ± 0.4bc	39.7 ± 1.3abc
	STFFOF	64.9 ± 2.6b	12.1 ± 0.5a	5.7 ± 0.0a	1.0 ± 0.0a	25.5 ± 0.3a	38.7 ± 1.9abc
2021	Blank	57.5 ± 3.3bc	12.2 ± 1.4a	5.4 ± 0.0b	0.8 ± 0.1a	22.6 ± 0.2b	43.6 ± 0.5a
	Con.	48.9 ± 2.5cd	8.3 ± 0.3b	5.0 ± 0.0c	0.9 ± 0.1a	19.8 ± 0.4c	36.0 ± 0.3c
	STFFOF	81.7 ± 5.1a	11.9 ± 0.7a	5.6 ± 0.1a	0.9 ± 0.0a	24.7 ± 0.8a	41.9 ± 1.3ab
ANOVA results							
	Treatment	<0.001	0.003	<0.001	0.649	<0.001	0.202
	Year	<0.001	0.857	0.984	0.159	0.997	0.174
	Treatment × year	0.031	0.946	0.150	0.690	0.160	0.025

Blank, no fertilizer treatment; Con., control; STFFOF, soil-testing formula fertilization using organic fertilizer. Mean values ± standard errors (n = 3) in the same column followed by different letters are significantly different (P ≤ 0.05). The values of ANOVA results in bold show significant treatment effects at P ≤ 0.1.

### Mineral elements of rice stem

Compared with that in the control, STFFOF reduced the concentrations of SiO_2_, K, Ca, and Mg in the basal internodes by 38%**, 9.5%*, 32.5%*, and 29.8%*, respectively, but increased those of N and P by 76.3%** (averaged over the last 2 years), with concentrations of SiO_2_, K, Ca, and P showing a significant difference between years (**Table 4**). The correlation analysis (**Table 5**) showed that the stem lodging rate was only negatively correlated with the concentration of Ca in internodes but significantly positively correlated with those of N and P, without correlation with other elements.

**Table 4:** Effects of soil-testing formula fertilization using organic fertilizer for 11 years on the mineral elements of rice stem.

Year	Treatment	SiO_2_ (g/kg)	N (mg/g)	P (mg/g)	K (mg/g)	Ca (mg/kg)	Mg (mg/kg)
2020	Blank	69.1 ± 4.6a	4.1 ± 0.5c	2.6 ± 0.3b	3.4 ± 0.2c	0.9 ± 0.1b	7.2 ± 0.4a
	Con.	51.0 ± 2.4b	7.2 ± 0.5bc	4.4 ± 0.3b	7.0 ± 0.4bc	1.1 ± 0.0bc	9.5 ± 0.5a
	STFFOF	27.7 ± 2.5d	12.3 ± 0.9a	4.9 ± 0.4b	6.0 ± 0.2bc	1.0 ± 0.2b	6.9 ± 0.6a
2021	Blank	41.3 ± 2.9bc	7.0 ± 0.2bc	4.7 ± 1.0b	10.1 ± 1.4ab	1.6 ± 0.2b	6.6 ± 0.9a
	Con.	45.3 ± 0.6b	8.4 ± 0.4b	5.0 ± 0.5b	13.8 ± 1.7a	2.5 ± 0.2a	9.7 ± 0.7a
	STFFOF	32.0 ± 1.0cd	15.2 ± 1.6a	11.7 ± 1.8a	12.8 ± 1.3a	1.4 ± 0.3bc	6.6 ± 1.1a
ANOVA results							
	Treatment	<0.001	0.000	0.003	0.043	0.015	0.013
	Year	0.004	0.012	0.004	<0.001	<0.001	0.781
	Treatment × year	0.001	0.637	0.033	0.998	0.045	0.916

Mean values ± standard errors (n = 3) in the same column followed by different letters are significantly different (P ≤ 0.05). The values of ANOVA results in bold show significant treatment effects at P ≤ 0.1.

Blank, no fertilizer treatment; Con., control; STFFOF, soil-testing formula fertilization using organic fertilizer.

**Table 5:** Relationships among the chemical indicators of rice stem under soil-testing formula fertilization using organic fertilizer (STFFOF) for 11 years.

Index	SLR	LI	BM	SiO_2_	N	P	K	Ca	Mg	CW	Lignin	Cellulose	SS
LI	-0.076												
BM	0.498+	0.624^a^											
SiO_2_	-0.464	0.540+	-0.007										
N	0.730^b^	-0.197	0.340	-0.746^b^									
P	0.644^a^	0.054	0.366	-0.316	0.787^b^								
K	0.133	0.496	0.620^a^	0.109	0.213	0.513+							
Ca	-0.628^a^	0.503	0.237	0.246	-0.244	-0.114	0.521+						
Mg	-0.269	0.568+	0.188	0.619^a^	-0.475	-0.281	0.345	0.188					
CW	-0.015	0.011	-0.107	0.098	-0.164	-0.026	0.363	-0.045	0.345				
Lignin	-0.069	0.466	0.602^a^	0.094	0.146	0.418	0.849^b^	0.748^b^	0.093	-0.042			
Cellulose	0.485	0.165	0.393	-0.078	0.506+	0.775^b^	0.755^b^	-0.006	0.074	0.456	0.458		
SS	-0.550+	0.356	0.122	0.299	-0.169	0.086	0.376	0.815^b^	0.070	-0.333	0.668^a^	-0.059	
Starch	-0.611^a^	0.003	-0.117	0.167	-0.142	0.045	0.303	0.757^b^	-0.147	-0.276	0.638^a^	0.010	0.808^b^

The P-values in bold show significant correlations at P <0.01. N = 12.

SLR, stem lodging rate; LI, lodging index; BM, bending moment; CW, cell wall; SS, soluble sugar.

a Statistically significant correlations at P < 0.05.

b Statistically significant correlations at P < 0.01.

### Carbohydrate content

Compared with those in the control, the concentration of cell wall, lignin, and soluble sugar decreased by 1.5%, 4.7%, and 11.1%, respectively, on average in 2020 and 2021 upon STFFOF, with a significant difference of these parameters except for cell wall between years (**Table 6**). The correlation analysis (**Table 5**) showed that the stem lodging rate was negatively correlated with the concentration of nonstructural carbohydrates, such as internode starch and soluble sugar, but had no significant correlation with that of structural carbohydrates, such as cellulose and lignin.

**Table 6:** Effects of soil-testing formula fertilization added organic fertilizer (STFFOF) for 11 years on carbohydrate content of rice stem (%).

Year	Treatment	Cell wall	Lignin	Cellulose	Soluble sugar	Starch
2020	Blank	83.4 ± 2.1a	17.2 ± 0.4b	44.4 ± 0.5a	8.8 ± 0.7abc	4.2 ± 0.4ab
	Con.	81.2 ± 1.5ab	17.0 ± 0.7b	44.3 ± 0.3a	8.0 ± 0.3bc	2.8 ± 0.3bc
	Tr.	83.4 ± 1.7a	15.7 ± 0.5b	42.8 ± 2.2a	6.3 ± 0.7c	2.5 ± 0.2c
2021	Blank	72.8 ± 0.9b	24.2 ± 0.2a	62.3 ± 5.7a	11.5 ± 1.4a	4.7 ± 0.4a
	Con.	84.9 ± 3.9a	24.2 ± 0.6a	53.3 ± 10.0a	10.0 ± 1.0ab	3.7 ± 0.2abc
	OFT	80.3 ± 2.1ab	23.6 ± 0.5a	66.3 ± 7.5a	9.1 ± 0.4ab	3.4 ± 0.5abc
ANOVA results						
	Tr.	0.210	0.194	0.693	0.091	0.015
	Year	0.165	<0.001	0.012	0.012	0.048
	Treatment × year	0.069	0.780	0.589	0.923	0.889

Blank, no fertilizer treatment; Con., control; STFFOF, soil-testing formula fertilization using organic fertilizer. Mean values ± standard errors (n = 3) in the same column followed by different letters are significantly different (P ≤ 0.05). The values of ANOVA results in bold show significant treatment effects at P ≤0.1.

## Discussion

### Severe stem lodging of rice prevented STFFOF’s long-term application

Based on the theory of soil-testing formula, different fertilization ratios should be used according to the soil quality in different areas—for instance, the most suitable fertilization formula was 50 kg N ha^-1^ of urea and 100 kg N ha^-1^ of organic fertilizer in rice fields in Shanghai as observed by Zhao et al. (2016). In southwestern China, Zhang et al. found that, compared with traditional farming methods, the type of contour tillage added organic matter reduced soil erosion by 14% (Zhang et al., 2016). The soil testing results indicated that 50% replacement of organic fertilizer and 50% reduction of pesticide had the best ecological and economic benefits (Wang et al., 2021). Our preliminary results showed that STFFOF was the best mode of fertilization application in this area compared with control as confirmed by the yield results. STFFOF increased the actual yield of rice averaging by 12.1% for 10 seriate years. Organic fertilizer posed a gradual effect on rice production probably due to the “accumulative effect” of organic fertilizer on improving soil. In the previous 3 years of STFFOF treatment, the STFFOF-induced yield fluctuation was very small with a slight increase of 6.5%. However, since the fourth year, the yield increase rate began to sharply grow. It increased to an annual average of 14.4%, up to 25.5% (2016, **Figure 3**), probably due to the improvement of soil ecological environment caused by the long-term application of organic matter.

It also had negative effects—for example, a test performed by Wang et al. showed that, in the Taihu Lake region, compared with the use of chemical fertilizers, clean agricultural measures, such as organic fertilizer use, reduced N losses by 19%–54% but also increased P losses by 45%–237% (Zhao et al., 2015). The long-term use of organic fertilizers may also cause heavy Zn or Cd pollution (Suzuki et al., 2009). Moreover, our present results show that the negative effect of STFFOF after 11 years was more serious. Since the ninth year, rice stem lodging worsened year by year. From 2020 to 2021, the annual average rate of visual stem lodging in paddy reached as much as 25.9 percentage points in comparison with control (**Figure 1A**), and the yield-increasing effect caused by STFFOF also decreased year by year. The actual yield of rice lowered instead by 26.1% upon STFFOF treatment with only 9.4% higher than that of the blank in 2021 (**Figure 3**). In fact, the theoretical yield still increased by 22.9%** (**Figure 1C**). These findings indicated that the risk of rice visual stem lodging induced by excessive exploration mode of productivity technology of paddy field, such as STFFOF, was great and must be prevented in the future.

### STFFOF-induced changes in N and P in basal internodes may mainly contribute to the stem lodging of rice

In this study, the concentration of N in the basal internodes was also significantly increased by 76.3%** owing to STFFOF (**Table 4**); however, it did not reduce the stem diameter, stem wall thickness, the cross-sectional area of the stems, and the pushing resistance of the rice plants. The application of organic fertilizer in STFFOF increased the stem diameter, stem wall thickness, cross-sectional area, and bending resistance of basal internodes by 11.4%**, 4.3% ns, 24%**, and 49.6%** (**Table 3**), respectively, but significantly reduced the lodging index by 28.2% (**Figure 1D**). The pushing resistance per plant and per stem were also reduced by 35.9%** and 44.8%** (**Table 2**), respectively, resulting in an increase of 25.9 percentage points (**Figure 1A**) in the visual actual stem lodging rate. Moreover, it did not cover the root lodging of rice plants in the field.

Interestingly, under STFFOF, the visual stem lodging rate in paddy fields was positively related to the breaking resistance (*P* = 0.695*, **Table 1**) and stem diameter (*P* = 0.654*, **Table 1**) of basal internodes, showing a significant or extremely significant negative correlation with pushing resistance per plant (*P* = −0.716*, **Table 1**)/culm (*P* = −0.854**, **Table 1**) but presented no significant correlation with lodging index (*P* = −0.076 ns, **Table 1**). These results indicated that (1) the visual stem lodging rates in paddies could be accurately speculated *via* the pushing resistance per plant or stem rather than the bending resistance or lodging index of internodes, especially under the STFFOF and (2) the increase in the concentration of N in basal internodes might not be the only direct or main reason of large-scale stem lodging in paddy fields, although the stem lodging rate displayed a significant positive correlation with the N concentration in basal internodes (*P* = 0.730**, **Table 1**). We observed that the increase in N concentration inhibited the absorption of other elements, *e*.*g*., STFFOF reduced the concentration of SiO_2_, K, Ca, and Mg of stem by 38%**, 9.5%*, 32.5%*, and 29.8%* (**Table 4**), respectively, averaged across 2020 and 2021 in this report, and the stem lodging rate was negatively correlated with that of Ca (*P* = −0.628*, **Table 5**). Excessive output induced by the long-term application of organic fertilizer in STFFOF mode overdrew arable and destroyed the ecological resilience of paddy fields, leading to uneven accumulation of N and other elements in plants, and further presented the evident “antagonism” phenomenon among mineral elements, finally increasing the brittleness of the stem and the occurrence of stem lodging (Kong et al., 2013; Chen et al., 2020; Zhang et al., 2021). What is the mechanism of unbalanced accumulation of mineral elements caused by STFFOF? Why did the stem lodging in this research only occur until the ninth year after STFFOF? No relevant report was published.

Otherwise, P fertilizer was also added into the STFFOF treatment, which may be the direct reason for the significant increase in P concentration in internodes (76.3%**, **Table 4**). Interestingly, a significant positive correlation between stem lodging rate and stem P (*P* = 0.644*, **Table 5**) was observed. It was contrary to the report that similar P application enhanced lodging resistance in rice (Chen et al., 2020). This result indicated that the unbalanced accumulation of mineral elements may not be the most important cause for stem lodging; the large-scale lodging of rice caused by STFFOF might relate to other more important factors.

### The decrease in non-structural carbohydrate concentration might be another important reason for stem fragility

Predecessors thought that structural carbohydrates, such as cellulose (Crook and Ennos, 1995) and lignin (Tripathi et al., 2003; Cui et al., 2018), in rice stems were closely related to the pushing resistance of rice. Higher proportions of structural carbohydrates enhanced stem stability in crops (Zhang et al., 2014a; Nguyen et al., 2016), but some scholars also reported no clear relationship between lodging and carbohydrate content (Kashiwagi et al., 2008; Gomez et al., 2018). This study also showed no evident relationship between lodging and carbohydrate concentration (**Table 5**), which agreed with previous reports (Kashiwagi et al., 2008; Gomez et al., 2018). The visual stem lodging rate of rice plant was instead closely related to the concentrations of starch (*P* = −0.611*, **Table 5**) and soluble sugar (*P* = −0.550+, **Table 5**) in the basal internodes, which was also observed by predecessors (Yang et al., 2011). Fumigating high CO_2_ concentration during the growth period increased the concentration of starch as suggested by Zhao et al. (2019), resulting in the enhanced pushing resistance of rice. However, they believed that the increased pushing resistance was mainly due to the increased culm density induced by the high concentration of nonstructural carbohydrates. This long-term located field experiment showed that the concentration of nonstructural carbohydrates, such as starch (*P* = −0.105 ns) and soluble sugar (*P* = −0.034 ns) in basal internodes, was unrelated to stem density. However, the stem lodging rate of rice plants also increased, indicating that the effects of non-carbohydrates on stem lodging were not achieved by increasing stem density under STFFOF. Similar evidence regarding this relationship between starch and stem density under other conditions was also observed in this study (Kashiwagi et al., 2008). We recognized that organic fertilizer application altered the assimilated distribution within plant organs, with a lower proportion of nonstructural carbohydrates stored in stems, probably reducing the stem strength and increasing the brittleness and lodging susceptibility of rice stem because most lodging observed in our paddy was stem fracture rather than bending or buckling (Abstract figure). Why did the concentration of nonstructural carbohydrate in plant stems exhibit evident “accumulation” phenomenon after the application of STFFOF that lasted for 11 years? Organic fertilizers contain large numbers of beneficial microorganisms. After the application of fertilizer to the soil, these microorganisms can further grow and reproduce under suitable conditions and directly provide certain elements, hormones, and various enzymes to the crops, thereby increasing the nutrients required by the crops; the nutrients required for the crop are indirectly provided by microbial reactions (Wang et al., 2019). This effect of organic fertilizer on the soil ecology is a long process. We speculated that this soil ecology mainly contributed to assimilate distribution within plant organs.

A higher number of tillers per unit land area theoretically increase population density, and heavier panicles impose more pressure on stems; both factors would increase lodging risk (Kong et al., 2014; Hirano et al., 2017). We also found that STFFOF significantly increased the weight per ear by 18.4% (2021, **Table 5**). However, an inappropriate result was collected, that is, no significant correlation was found between the stem lodging rate and weight per ear when the dry weight per ear (2020) and wet weight per ear (2021) were analyzed because the former one was only analyzed in 2020. However, when the simulated data of fresh weight per ear in 2020 according to the average ear water rate in 2021 were adopted, the weight per ear was significantly associated with the stem lodging rate (*P* = 0.824**, *n* = 12). Moreover, the analysis data results in 2021 showed the same significant positive relationship (*P* = 0.876*, *n* = 6). Meanwhile, the SPAD in the same growth stage increased by 21%** (**Table 2**) after 11 years of continuous STFFOF treatment, which was in concurrence with our observation in paddies (**Figure 2**). The finding indicated that STFFOF greatly prolonged the growth period of rice, further resulting in a larger weight per ear in the same period than the control. In addition, the growth period of the blank for 11 years was the shortest among the three treatments, and the weight per ear and SPAD in the same period were only 90.0% and 59.2% of that in the STFFOF treatment, respectively; thus, no lodging phenomenon was observed in the blank. Therefore, we speculated that ear weight may contribute to another cause to the large-scale stem lodging of rice in paddies.

In summary, STFFOF significantly increased the visual stem lodging rate of rice plants by 81.1%*, thereby outweighing the STFFOF-induced increase in grain yield. The in-depth study found that STFFOF significantly reduced the concentration of Ca and starch in the basal internodes and evidently increased those of N and P, as well as the weight per ear in the same period, without effect on the concentration of lignin, cellulose, and cell wall. The significant relationship presented the increase in weight per ear and the concentration of N and P in basal internodes, especially the significant decrease in that of starch. This finding might directly lead to the brittleness increase in the internodes and then further cause the large-area stem lodging and severe yield loss of rice.

## Data availability statement

The original contributions presented in the study are included in the article/supplementary material. Further inquiries can be directed to the corresponding author.

## Author contributions

FZ: conceptualization, methodology, and writing—original draft. FL: investigation. JZ: investigation. XS and YW: funding acquisition. LJ: formal analysis and writing—review and editing. JH: investigation. FB: formal analysis and visualization. GW: funding acquisition. BC: conceptualization, methodology, and writing—review and editing. All authors contributed to the article and approved the submitted version.
